# Ambiguity tolerance in organizations: definitional clarification and perspectives on future research

**DOI:** 10.3389/fpsyg.2015.00344

**Published:** 2015-04-28

**Authors:** David L. McLain, Efstathios Kefallonitis, Kimberly Armani

**Affiliations:** ^1^School of Business, State University of New York at Oswego, Oswego, NY, USA; ^2^Metro Center, State University of New York at Oswego, Syracuse, NY, USA

**Keywords:** ambiguity tolerance, uncertainty, complexity, unfamiliarity, entropy, illogical stimuli, decision making, cognition

## Abstract

Ambiguity tolerance is an increasingly popular subject for study in a wide variety of fields. The definition of ambiguity tolerance has changed since its inception, and accompanying that change are changes in measurement and the research questions that interest researchers. There is a wealth of opportunity for research related to ambiguity tolerance and recent advances in neuroscience, measurement, trait research, perception, problem solving, and other fields highlight areas of interest and point to issues that need further attention. The future of ambiguity tolerance research is promising and it is expected that future studies will yield new insights into individual differences in reactions to the complex, unfamiliar, confusing, indeterminate, and incomplete stimuli that fall within the conceptual domain of ambiguity.

## Introduction

The study of ambiguity tolerance continues to flourish after more than 65 years of study and is finding new appeal among scholars in a widening array of fields of inquiry. Interestingly, the original study of the concept of ambiguity tolerance focused on sociological correlates rather than cognitive psychological reactions to ambiguous stimuli, the focus of most current research. Early scholars held divergent views on an individual’s orientation toward ambiguity. One view held that individuals who could not arrive at clear and conclusive interpretations of situations were psychologically weaker than other people (Jaensch, 1938). By contrast, a second view, introduced by Frenkel-Brunswik (1948), referred to such people as ambiguity intolerant, and promoted a virtuous conceptualization of tolerance for ambiguity. Much of the early research related to ambiguity tolerance concerned individuals’ reactions to other people and cultures, and their perceived differences. Extending this view, a group of social psychologists advanced the argument that ambiguity intolerance was a personality trait that explained a variety of negative social attitudes and behaviors within the general domain of prejudice against people unlike themselves (e.g., Adorno et al., 1950). Empirical evidence for the latter argument was equivocal (Ray, 1990) and the concept of ambiguity tolerance gradually underwent refinement to become more narrowly focused and become grounded in more psychologically-sound reasoning. This refinement was accompanied by emerging interest in other potential consequences of an individual’s inherent orientation toward ambiguous stimuli. Eventually, a conceptual definition of ambiguity tolerance emerged that was restricted to individual differences in cognitive reaction to stimuli perceived as ambiguous (McLain, 1993), helped along by conceptual focus (Budner, 1962) and greater rigor in measurement (e.g., Rydel and Rosen, 1966; MacDonald, 1970; Norton, 1975). Today, researchers consider ambiguity tolerance to be an individual difference that predicts short and long-term reactions to a spectrum of situational characteristics relevant in a wide variety of life contexts and outcomes (Furnham and Marks, 2013).

This paper has two goals. First, the conceptual definition of ambiguity tolerance will be discussed. This discussion is intended to clarify a cognitive and individual differences meaning for the construct and support the inclusion of ambiguity tolerance in the widest array of future research studies. The second goal of this paper is to list areas for future research that have not been fully explored, but which have the promise of providing useful insights into cognitive and behavioral phenomena. This second goal will emphasize research in the organizational sciences and identify some areas of current and promising interest among scholars while retaining a manageable scope.

## Definition

Across the spectrum of definitions, both conceptual and operational, there is general agreement that ambiguity tolerance represents a relatively stable individual difference. However, over the decades of its existence as a subject of scholarly interest, ambiguity tolerance has been subject to multiple definitional orientations including its initial conceptualization as a trait with sociological implications (Frenkel-Brunswik, 1948); a multi-dimensional personality trait (e.g., Budner, 1962); and a descriptor of organizations and national cultures (reviewed in Furnham and Ribchester, 1995). Frenkel-Brunswik (1949) identified intolerance of ambiguity as a personality trait and emphasized its role in forming judgments of other people, identifying ambiguity intolerant people as “those with a tendency to resort to black white solutions, to arrive at premature closure as to valuative aspects, often at the neglect of reality, and to seek for unqualified and unambiguous over-all acceptance and rejection of other people.” Frenkel-Brunswik (1949) considered ambiguity tolerance a core variable “in the emotional and cognitive orientation of the individual toward life.” This sweeping conceptual description was redefined by Budner (1962), who focused his definition more narrowly and specifically on intolerance of ambiguity without automatic extension to sociological or personality correlates. Budner (1962) wrote, “Intolerance of ambiguity may be defined as ‘the tendency to perceive (i.e., interpret) ambiguous situations as sources of threat’ and tolerance of ambiguity as ‘the tendency to perceive ambiguous situations as desirable’.” For the purposes of this paper, the definition of ambiguity tolerance will be restricted to the domain of an individual’s cognitive sensitivity to ambiguous stimuli, with roots in neurophysiological function and with expressions in psychology and choice. For in-depth reviews of the many definitions of ambiguity tolerance, the reader is encouraged to read excellent discussions by Furnham and Ribchester (1995) and Furnham and Marks (2013).

To better understand ambiguity tolerance, it is useful to distinguish it from ambiguity. Ambiguity is a perception and is a function of the information received regarding a focal stimulus. Ambiguity tolerance is an individual’s systematic, stable tendency to react to perceived ambiguity with greater or lesser intensity. Some forms of ambiguity have secondary attractiveness; for example, a mysterious character in a story can be both anxiety inducing, because he or she is ambiguous, and at the same time intriguing, because of the anticipated satisfaction that will result when and if the mystery is solved and the ambiguity is eliminated. Ambiguity can also obscure opportunities with favorable potential. Consequently, ambiguity can produce both aversion and attraction, and these reactions are contingent upon how perception of the ambiguous stimulus interacts with information about the context and anticipated outcomes.

Different approaches to defining ambiguity appear in different fields of inquiry. Ellsberg (1961) provides a description of ambiguity from the perspective of economics by relating ambiguity to risk and uncertainty. Ambiguity, writes Ellsberg, is “…a quality depending on the amount, type, reliability, and ‘unanimity’ of information, and giving rise to one’s degree of confidence in an estimate of relative likelihood (of outcomes).” Drawing on Ellsberg’s work, Einhorn and Hogarth (1986) conducted experiments which strengthened understanding of the relationship between ambiguity and cognition. Ambiguity has also been defined in cognitive terms as a lack of distinction between multiple interpretations or meanings in a situation (e.g., Weick et al., 2005). Such a situation may exhibit characteristics associated with multiple situations but does not clearly fall within one characterization—possibly having unexpected or illogical characteristics making it difficult to decidedly characterize the situation. Ambiguity has also been described as that which is different from the familiar with emphasis on people and cultures (e.g., Adorno et al., 1950).

Because ambiguity tolerance is an individual difference construct describing how individuals process, interpret, and react to information, it is worthwhile to examine it from a neurological perspective. The brain reacts to stimuli as informed by sensory mechanisms. Therefore, the information that is received comes from tactile, auditory, visual, olfactory, and taste sensors and is transmitted to the brain. The dorsolateral prefrontal cortex is home to the executive controller that coordinates the receipt and processing of that information, therefore, ambiguity challenges this part of the brain. Ambiguity is a situation that presents too little necessary information to be clearly understood and classified into a mental model that enables choice and action (Hsu et al., 2005). When available information fails to support a conclusive interpretation and the situation is sufficiently important that the fully developed, healthy brain wants a conclusive interpretation, perhaps to identify an appropriate behavioral response, anxiety (stress) is generated. The lack of information, therefore, induces anxiety which initiates cognitive effort to resolve the ambiguity. The degree that anxiety is generated in response to an indefinite collection of data received about a situation is ambiguity tolerance and is a stable trait of the individual. The intensity of the reaction to perceived ambiguity indicates the individual’s ambiguity tolerance.

Elaborating and detailing a definition of ambiguity tolerance may be helped by further examining the concept of tolerance, or sensitivity, and describing the types of stimuli that fall within the domain of ambiguity. Sensitivity refers to the degree of reaction an individual has in response to sensing an ambiguous stimulus. This sensitivity can, in theory, range from extreme aversion to extreme attraction. Extreme aversion is manifest in either absolute rejection of the stimulus or the maximum motivational arousal intended to reduce the ambiguity to an acceptable level. Extreme attraction is, conversely, a maximal motivation to seek and embrace ambiguity and enjoy its novelty, complexity, and insolubility resulting in high levels of curiosity and positive affect. Cognitively, sensitivity manifests in two ways: the individual can experience fear or anxiety due to perceived ambiguity (Hirsh et al., 2012). Both can motivate the search for clarity while anxiety may manifest as excitement in situations of attraction (Levi, 1965).

The perception of ambiguity can be threatening because it obscures potential harm (Budner, 1962). Evidence from neurological research indicates that ambiguity induces anxiety more directly than fear. Ambiguity hinders choice because desirable alternatives are difficult or impossible to distinguish from undesirable members of the feasible set of options (Hirsh et al., 2012). In an ambiguous situation, behavioral choices cannot be linked to predictable outcomes. Thus, alternative conceptualizations of cognition-behavior-outcome relationships must be developed and examined until predictability can be achieved. Individuals are generally averse to ambiguity whenever the ambiguous situation presents the possibility of tangible, negative consequences. Conversely, curiosity and attraction to ambiguity are more likely to occur when the domain of potential outcomes excludes harm (McLain, 2009). If threat is minimal, ambiguity can be attractive, as when we seek to watch a murder mystery where the clues point to several individuals and we do not have enough information to identify the true culprit. In such cases, we are attracted to the ambiguity, even though it still generates anxiety, and we engage with the story until the ambiguity is resolved and our curiosity is satisfied. There is no personal threat to us when we experience this ambiguity.

Threat is one reason that ambiguity is seldom attractive to people. In many circumstances, ambiguity hides a potential threat and is therefore undesirable. At first glance, thrill-seeking and risk-taking behavior might appear to conflict with the claim that threat is undesirable. Risk-taking propensity and ambiguity tolerance are positively related traits and both traits predict behavior toward situations that are not perfectly predictable (McLain, 1993). However, risk-taking does not require the perception of ambiguity. Risk-taking is often associated with situations in which there is the potential for harm but little or no ambiguity about which options are available or what harm might occur. In such situations, the individual may have an unambiguous expectation that he or she will not experience harm despite the real danger that energizes that person (Farley, 1986; MacCrimmon and Wehrung, 1988).

Multiple types of stimulus characteristics have the potential to be ambiguous (McLain, 1993). These are characteristics that pose the challenge of indistinguishability among cognitive and behavioral interpretations. A lack of familiarity, also called new or novel, is one such stimulus characteristic. Until information is gathered that enables the development of predictive understanding, unfamiliarity is associated with the anxiety of ambiguity. Complexity is distinct from unfamiliarity, but can induce the same perception of ambiguity because it takes time and effort to understand a complex stimulus. Insufficient information needed to understand a situation is also ambiguous if it bars a well-distinguished understanding of the situation. In the same way, any stimulus characteristic that results in situational interpretations being indistinguishable is ambiguous. These characteristics include illogic, opacity or translucence, conflicting situational elements (like rain on a sunny day), unpredictable dynamics, and any other characteristic that impedes a clear interpretation and behavioral response (McLain, 2009). Another characteristic common to most ambiguous situations is that more information will reduce it. Indirectly, this means that time attenuates ambiguity—with time comes more information, which affords the opportunity for greater understanding and less ambiguity.

It is also valuable to distinguish ambiguity tolerance from its cognitive and behavioral consequences. Being strongly averse to ambiguity is part of an individual’s ambiguity tolerance. Avoiding situations perceived as ambiguous is a consequence of that trait. A wide range of reactions are possible consequences of an individual’s ambiguity tolerance, such as career avoidance, delayed decision making, inaccurate choices, errors made in attributing causality, and other reactions ranging from the general to the situation-specific. These reactions explain the relevance and importance of the construct and are reasons for its interest among researchers.

The operational definition of ambiguity tolerance began with laboratory measurements of reactions to various stimuli (Jaensch, 1938; Frenkel-Brunswik, 1949) but a widely-accepted measure did not appear until Budner’s (1962) 16-item, paper-and-pencil scale. Budner’s (1962) scale items identified ambiguity intolerance with a set of four possible reactions (phenomenological denial or submission and operative denial or submission) and three stimulus characteristics (novelty, complexity, or insolubility). Although Budner’s (1962) measure did not have strong psychometric properties, it has been widely used and helped further the study of ambiguity tolerance by improving the psychological definition of ambiguity intolerance and accompanying the conceptual definition with an operational definition. Subsequent operational advances have been made with paper-and-pencil measures that attempt to improve psychometric properties and increase the attention paid to stimulus types that are perceived as ambiguous (McLain, 1993). Further advances are expected as theory draws on increasing knowledge of neurological responses to ambiguous stimuli (Hirsh et al., 2012).

### Personality and Trait Correlates

A number of traits related to ambiguity tolerance are discussed in the literature. Among those traits are those that describe stable reactions to stimuli closely related to ambiguity including stimuli that are risky or uncertain. Risk-taking propensity is the orientation of the individual toward risks and there are different incarnations of this propensity associated with different types of risk; for example, financial, professional, or health risks. Risk is broadly defined as the probability and magnitude of an undesirable outcome to a situation (Kaplan and Garrick, 1981; Cohrssen and Covello, 1999). It is possible to identify possible outcome states and attach quantitative likelihoods to each state if a situation is properly called “risky.” The lack of information specifying which state will obtain makes a risky situation threatening and stressful. Risk-taking propensity is, therefore, conceptually related to ambiguity tolerance and this has been confirmed by research as a strong, positive correlation between risk-taking propensity and ambiguity tolerance (e.g., McLain, 1993). This close relationship extends to more than risk orientation in business decision making. Ambiguity tolerance also correlates positively with an individual’s orientation toward physical risks, measured as sensation seeking (Zuckerman et al., 1964). Other related traits which have been or should be examined for their relationship with ambiguity tolerance, because they indicate sensitivity to information conditions, include uncertainty avoidance, need for closure, and curiosity. There are several avenues open to research of relationships between ambiguity tolerance and other personalities and traits.

### Perceptual Correlates

Just as there are personality traits related to ambiguity tolerance, there are also perceptual correlates. Because of the nature of the stimuli and the threats those stimuli present, both perceived risk and perceived uncertainty are likely to be influenced by the perceiving individual’s ambiguity tolerance. Perceived risk is the most often studied cognition in the risk psychology literature (e.g., Slovic, 1987). Perceived environmental uncertainty is an important input to strategic decision making, and because it is negatively correlated with an individual’s ambiguity tolerance, it confounds normative strategic theory with an individual difference (Lorenzi et al., 1981). Another example where ambiguity tolerance may be a relevant moderating influence is research and theory of sensemaking (Weick, 1995). The initiation of the sensemaking process is situational ambiguity (Weick et al., 2005), making ambiguity tolerance a possible factor in starting this process and an area of inquiry that may interest future researchers.

## Opportunities for Research Concerning Ambiguity Tolerance

Despite many decades of research concerning ambiguity tolerance, much remains to be learned. Recent advances in fields such as neuroscience have raised new questions about ambiguity and ambiguity tolerance and have clarified existing understanding regarding interpretations and reactions to ambiguous stimuli (e.g., Smith et al., 2002; Rustichini et al., 2005). Further growth in scholarly interest is due at least in part to the development of theoretical perspectives on human behavior that begin with the premise that situational ambiguity is a critical influence or initiating condition antecedent to choice preferences and behaviors. In organizational studies, for example, ambiguity is a factor that determines suitable designs and policies for operation (Weick et al., 2005). An example of a potentially useful theoretical foundation for future ambiguity tolerance research might be the Entropy Model of Uncertainty developed by Hirsh et al. (2012) which is grounded in neuroscience and information theory. According to this model, ambiguity, uncertainty, or other form of inadequate situational information motivates the individual to reduce the ambiguity to a manageable level. Within this framework, ambiguity tolerance may (1) play the role of a moderator of motivation to reduce perceived ambiguity, (2) govern the degree of conflict experienced between perceptual and behavioral affordances, (3) be substituted by goal clarity and specificity, and (4) amplify or attenuate the anxiety experienced when exposed to ambiguous stimuli. This framework illuminates the fundamental nature of ambiguity and ambiguity tolerance in cognition and behavior and helps explain why ambiguity tolerance is a subject of continuing interest in several fields of study. This perspective also suggests pathways for future research.

Currently, studies appear in such specific and diverse areas as consumer perceptions (Hazen et al., 2012), nursing leadership (reviewed by Brady Germain and Cummings, 2010), medical education (Luther and Crandall, 2011), professional decision making (discussed in Appelt et al., 2011), business communication (Russ, 2012) adolescent safety (Tymula et al., 2012), and several other areas where differential reactions to the perception of stimulus information may influence interpretation and behavioral choice. In addition, promising research is being conducted at the neurological level where the links between brain physiology, function, and the perception of ambiguous stimuli are becoming increasingly clear (e.g., Schick et al., 2013). Regardless of these advances, there is considerable work yet to be done in studying ambiguity tolerance as well as considerable interest in doing so.

One convenient way to classify ambiguity tolerance research, like research concerning other traits, is to group studies into three categories: measurement, correlates, and predictive research. The measurement of ambiguity tolerance has a long history and has been addressed by several scholars including Budner (1962), Rydel and Rosen (1966), MacDonald (1970), Norton (1975), and McLain (1993, 2009). Excellent reviews of the measurement literature have been published by Furnham and Ribchester (1995) and Furnham and Marks (2013). However, there is still work to be done regarding measurement. Although measures exist in several languages, there is a need for ambiguity tolerance measures in languages where there is not yet a measure. Many societies and cultures present interesting research questions but the lack of suitable measures in the missing languages makes such questions unanswerable.

A further need for measurement research arises due to advances in the understanding of brain function. Those advances argue for the development of measures that are cognizant of the individual’s neurological response to ambiguity (Hsu et al., 2005). We need measures that are directly constructed from this increased neurological understanding and that validly estimate individual differences in this response. Such research offers a possible avenue to measures of ambiguity tolerance that improve on the paper-and-pencil measures that now dominate related research. A related need is the identification of types of situations that initiate the pattern of brain activity most associated with response to ambiguity.

Among the correlates of ambiguity tolerance, there is considerable opportunity for research that identifies new correlates and links them through common characteristics. Finally, a broad need for additional research of the phenomena predicted by ambiguity tolerance exists. There are many areas in which there are opportunities for investigation and it is reasonably safe to say such opportunities exist in all areas of social and psychological science. These vary from the effect of cross-cultural environments (Caligiuri and Tarique, 2012; Bakir et al., 2015), to managerial implications (Wangrow et al., 2014), the health services field (Carleton, 2012) and education (Caulfield et al., 2014; Hancock et al., 2014).

Ambiguity tolerance may prove to be a useful variable for study in many fields of inquiry because of its theoretical role as a moderator of relationships between situational information and cognitive and behavioral reactions. For example, because ambiguity is theorized as a condition of sensemaking (Weick et al., 2005), ambiguity tolerance may moderate the relationship between an ambiguous situation and the initiation of sensemaking activity. This implies that ambiguity tolerance may not only vary among individuals in terms of reactivity to perceived ambiguity, but might serve to set the threshold over which sensemaking is motivated. The functional relationship between ambiguity tolerance and the threshold of reaction to situational ambiguity deserves study.

Another domain in which ambiguity tolerance may provide valuable insights is in the connection of memories to decision making. According to Reyna and Brainerd’s (1995) formulation of Fuzzy Trace Theory, interpretations and decisions regarding a situation are determined by a few facts that bolster desired interpretations and create, not a clear picture, but a “gist” of the situation. If so, this perspective suggests ambiguity is overcome using a gist and that ambiguity is not a large barrier to decision making in ambiguous situations. The ease with which that gist interpretation is formed may be influenced by aversion to ambiguity, that is, ambiguity tolerance, raising an issue for future research. An earlier theory regarding “Garbage Can” Decision Making suggests ambiguity about aspects of decision making shapes decisions in a number of ways including the adoption of solutions that were formulated before the problem situation arose (Cohen et al., 1972). In Social Information Processing Theory (Salancik and Pfeffer, 1978), perceived ambiguity motivates a worker to seek information from relevant others, such as coworkers, to provide interpretations of ambiguous work characteristics and form related and acceptable work attitudes. Ambiguity tolerance may serve to influence more or less of this social information seeking behavior and in that way influence the extent of conformity of the individual’s work attitudes to those that coworkers are believed to hold. In a related area, safety climate researchers argue that a worker’s perception of the safety attitudes of coworkers (i.e., safety climate) influences the worker’s own safety attitudes and behaviors (Zohar, 1980; Zohar and Luria, 2005). Ambiguity tolerance deserves investigation as a moderator of relationships between safety climate and the worker’s own safety attitudes and behaviors. Ambiguity tolerance may play a role in the phenomena that these, and other theories that depend on an assumption of situational ambiguity, try to explain.

Career choice is a situation often infused with ambiguity. Previous researchers have considered, for example, whether the choice of practice area among medical students is influenced by ambiguity tolerance (Matteson and Smith, 1977). The relative ambiguity of a career in home construction may be quite different from a career in the creative arts; therefore, future research might consider how the ambiguity tolerance of a job seeker influences choice of career based on the perceived ambiguity of the work associated with a career or of information available about that career option. Another avenue for study is whether the ambiguity of tasks performed by members of an occupation influences the attractiveness of that occupation to prospective entrants. Still other researchers seek to understand the motivations of entrepreneurs who, arguably, face greater ambiguity about their work than people who are not driven to create their own organizations (Begley and Boyd, 1988). Is ambiguity tolerance a core personality trait of entrepreneurs? Does ambiguity tolerance influence the stress or satisfaction associated with entrepreneurship?

Business planning and strategy present many opportunities for studying the effects of ambiguity tolerance. One area of study regards the related concept of uncertainty. Perceived environmental uncertainty research has revealed that the benefits of good strategic planning, which depend on an accurate assessment of uncertainty in the environment of the company, are often undermined because of inaccurate assessments (Lorenzi et al., 1981). This knowledge has not led to improvements in the accuracy of environmental uncertainty estimates. More up-to-date research is warranted to determine whether this problem continues and, if it does, how it might be addressed.

New product development is a business function of great importance. The development of new products is essential for businesses to grow and remain competitive. In spite of their importance, new products have failure rates on the order of 40% (Castellion and Markham, 2013) and higher. High failure rates are attributed in part to the uncertainty and risk inherent in developing new products. The amount of risk and uncertainty changes during the product development process. The front end of the process in particular, the “fuzzy front end,” is fraught with ambiguity and uncertainty. Previous research has suggested that success of products is related in part to how companies manage ambiguity (Frishammar et al., 2009). Unstructured processes are recommended to handle environmental uncertainty (Smith and Radeka, 2009). New to this world products pose the greatest ambiguity and risk but also promise the greatest reward (O’Connor and Rice, 2013). Ambiguity tolerance is related to new product portfolio decisions (McNally et al., 2009). Research shows a relationship between remanufactured products being of lower quality and consumers being less willing to pay for them (Hazen et al., 2012). Ambiguity tolerance is often cited as an important leadership quality (Cohen and March, 1986; Huber, 2003). Furthermore, creativity is an important characteristic of new product development team members. A connection between creativity and ambiguity tolerance has been proposed Lane and Klenke (2004). The preceding discussion suggests that future research examining the relationship between new product development team member ambiguity tolerance and new product development success is warranted.

Marketing researchers are interested in consumer responses to marketing methods and instruments. Ambiguity tolerance may moderate the influence of marketing information on consumer confusion and choice behavior (Mitchell et al., 2005). The large amount of information with which consumers are deluged presents ambiguity which can be confusing but also offers an opportunity to study relatively non-threatening ambiguity, due to the voluntariness of choice, and how it shapes consumer choice behavior. The study of ambiguity tolerance in marketing becomes of significant importance, considering the choices available to consumers among competing brands (Kefallonitis and Sackett, 2004). Current marketing and brand positioning techniques often confuse consumers (Walsh and Yamin, 2005; Wang and Shukla, 2013). Research that identifies the sensitivity of an individual to brand ambiguity and to the type and intensity of information cues that eliminate brand ambiguity and aid consumers in distinguishing one brand from another is needed (Sikkel, 2013). Such research would have both scholarly and applied value. As an example, it would provide differential advantage to a brand such as Coca-Cola, if it could identify and deliver brand messages that fell below the level of ambiguity to which most consumers are sensitive and might discourage a favorable attitude toward the message. To date, research evidence is limited to advertising, where probability markers affect consumer brand attitude with regard to less involving services (Banks and De Pelsmacker, 2014), but points to the value of such research (Richardson et al., 1996; Banks and De Pelsmacker, 2014). Further research highlights factors that affect tolerance in ambiguity such as: the effect of language differences (Dewaele and Wei, 2013; Grace, 2013), particularly as found in marketing (Alden et al., 1999), and various other cultural implications (Taylor, 2005, 2012). Expanding ambiguity tolerance research is needed particularly in the influence on differential reactions to well-established and emerging brands. Results cannot be generalized, as the research sample needs to include a multinational pool and reflect cultural and language differences.

### Conflict of Interest Statement

The Reviewer Xiaoyu Pu declares that, despite being affiliated to the same institution as the authors David L. McLain and Efstathios Kefallonitis, the review process was handled objectively and no conflict of interest exists. The authors declare that the research was conducted in the absence of any commercial or financial relationships that could be construed as a potential conflict of interest.
